# Reply to Gremese et al.: Statistical reasoning to evaluate treatment effects when data are collected with lack of design: Covid-19 experience

**DOI:** 10.1073/pnas.2103168119

**Published:** 2022-02-07

**Authors:** Clelia Di Serio, Pietro Cippà, Alessandro Ceschi, Paolo Ferrari

**Affiliations:** ^a^University Centre of Statistics in the Biomedical Sciences, “Vita Salute San Raffaele” University, 20132 Milan, Italy;; ^b^Biomedical Faculty, Università della Svizzera Italiana, 6900 Lugano, Switzerland;; ^c^Department of Medicine, Division of Nephrology, Ente Ospedaliero Cantonale, 6500 Bellinzona, Switzerland;; ^d^Faculty of Medicine, University of Zurich, 8006 Zurich, Switzerland;; ^e^Institute of Pharmacology and Toxicology, Ente Ospedaliero Cantonale, 6500 Bellinzona, Switzerland;; ^f^Department of Clinical Pharmacology and Toxicology, University Hospital Zurich, 8091 Zurich, Switzerland;; ^g^Clinical School, University of New South Wales, Sydney, NSW 2052, Australia

Gremese et al. (1) argue that “more data” are needed to assess the effects of treatments in patients with COVID-19.

A major lesson gained from the torrent of publications on COVID-19 (>200,000 in 2 y) concerns the importance of the quality rather than the quantity of data. Indeed, understanding the data-generating process is fundamental to evaluate data collected with lack of design in emergency protocols with no inclusion/exclusion criteria, no randomly selected cohorts, and, often, no adequate controls. In these situations, large amounts of data with poor data quality might magnify the effect of confounding bias instead of improving information.

Most published studies defined as “population based” investigate the effect of drugs in COVID-19 by computing odds ratios with controls extracted from public registries. However, proper “controls” should consist of infected disease-free subjects who are indeed hardly available. Even COVID-19 cohort studies may not really control for confounding effects, since the choice of cohorts in COVID-19 is also very critical. How can we evaluate the absolute effect on COVID-19 survival of nonsteroidal antiinflammatory drugs, antidiabetics, or anticoagulants by comparison with “administrative” controls or cohorts of patients with no information provided on their infective status or matched by all comorbidities?

This uncontrolled data frame should encourage researchers to find novel statistical methods for uncomplete study designs that account for the “unstructured” nature of the data.

In dealing with “real-world data,” increasing sample size may shrink the confidence intervals and amplify the impact of survey bias, an instance of big data paradoxes (2). Thus, the “amount” of data may not help in providing conclusive assessments on the combined effects of treatments in COVID-19 patients admitted in critical condition, mostly with several comorbidities and previous treatment protocols.

Even in the cited study on anticoagulants (direct oral anticoagulants [DOAC]) (3), out of 100,000 patients, there were only 360 hospital admissions for COVID-19 in patients on DOAC with atrial fibrillation (AF) versus two controls groups, one with AF and one with cardiovascular disease. Thus, any inference on possible effects of DOAC is not robust, with patients belonging to different populations with no correction for unbalanced comorbidities (kidney disease was threefold in the third cohort compared to the first).

In our paper (4), these considerations are placed within a “statistical thinking” perspective, “profiling” patients with respect to their survival driven directly by high-quality data and discovering what makes patients more likely to survive, “conditional” on the treatments.

We implemented different scenarios within a Bayesian perspective to evaluate dependence structure among covariates and the effect of different treatment combinations by means of posterior probability. This suggests the protective effect of renin–angiotensin–aldosterone system inhibitors (RAASi), removing doubts on discontinuing RAASi in hypertensive patients with COVID-19.

Randomized controlled trials (RCT) remain the standard to match potential confounders evenly between the groups. A recent multicenter RCT (5) showed that the RAASi telmisartan reduced morbidity and mortality in hospitalized COVID-19 patients, thus supporting our findings.

In conclusion, whenever the goal remains focused on generalizability of treatment effects, research should focus more on “good data” than “more data,” and on novel integrated statistical approaches that account for real study design to translate inferential conclusions in biomedical new findings.
